# Comparison of normalisation methods for surface-enhanced laser desorption and ionisation (SELDI) time-of-flight (TOF) mass spectrometry data

**DOI:** 10.1186/1471-2105-9-88

**Published:** 2008-02-07

**Authors:** Wouter Meuleman, Judith YMN Engwegen, Marie-Christine W Gast, Jos H Beijnen, Marcel JT Reinders, Lodewyk FA Wessels

**Affiliations:** 1 Bioinformatics and Statistics, Department of Molecular Biology, The Netherlands Cancer Institute, Amsterdam, The Netherlands; 2 Information and Communication Theory Group, Faculty of Electrical Engineering, Mathematics and Computer Science, Delft University of Technology, Delft, The Netherlands; 3 Department of Pharmacy & Pharmacology, The Netherlands Cancer Institute/Slotervaart Hospital, Amsterdam, The Netherlands

## Abstract

**Background:**

Mass spectrometry for biological data analysis is an active field of research, providing an efficient way of high-throughput proteome screening. A popular variant of mass spectrometry is SELDI, which is often used to measure sample populations with the goal of developing (clinical) classifiers. Unfortunately, not only is the data resulting from such measurements quite noisy, variance between replicate measurements of the same sample can be high as well. Normalisation of spectra can greatly reduce the effect of this technical variance and further improve the quality and interpretability of the data. However, it is unclear which normalisation method yields the most informative result.

**Results:**

In this paper, we describe the first systematic comparison of a wide range of normalisation methods, using two objectives that should be met by a good method. These objectives are minimisation of inter-spectra variance and maximisation of signal with respect to class separation. The former is assessed using an estimation of the coefficient of variation, the latter using the classification performance of three types of classifiers on real-world datasets representing two-class diagnostic problems. To obtain a maximally robust evaluation of a normalisation method, both objectives are evaluated over multiple datasets and multiple configurations of baseline correction and peak detection methods. Results are assessed for statistical significance and visualised to reveal the performance of each normalisation method, in particular with respect to using no normalisation. The normalisation methods described have been implemented in the freely available MASDA R-package.

**Conclusion:**

In the general case, normalisation of mass spectra is beneficial to the quality of data. The majority of methods we compared performed significantly better than the case in which no normalisation was used. We have shown that normalisation methods that scale spectra by a factor based on the dispersion (e.g., standard deviation) of the data clearly outperform those where a factor based on the central location (e.g., mean) is used. Additional improvements in performance are obtained when these factors are estimated locally, using a sliding window within spectra, instead of globally, over full spectra. The underperforming category of methods using a globally estimated factor based on the central location of the data includes the method used by the majority of SELDI users.

## Background

A wide range of mass spectrometry techniques is available, of which Matrix-Assisted Laser Desorption and Ionisation (MALDI) and Surface-Enhanced Laser Desorption and Ionisation (SELDI) [1] coupled with Time-Of-Flight (TOF) tubes are widely used for proteome screening. In both of these techniques a biological sample of interest, e.g., a serum sample, is applied to a plate or chip, left to incubate and subsequently co-crystallised with a matrix material. A laser is then fired at the co-crystallised mixture, causing it to desorb. The energy of the laser beam is transferred via the matrix to the analyte sample, thereby ionising it. An electrical field causes the desorbed and ionised material to fly through the TOF tube. Lower mass peptides travel faster through the tube than higher mass peptides, causing the former to arrive earlier at a detector placed at the end of the flight tube. Using a quadratic equation, the mass to charge ratio (*m*/*z*) of a peptide can be calculated. Because of the indirect (i.e., via the matrix) ionisation, laser desorption and ionisation is considered to be a "soft" ionisation method. As a consequence, most peptides will be single-charged, i.e., *z *= 1.

The main difference between MALDI and SELDI is that the latter normally uses a chip with a chromatographic surface, making the purification of the sample implicit. For MALDI, the purification needs to be done before application to the plate, for example through the use of chromatographic beads. Data resulting from mass spectrometry measurements usually contain a substantial amount of noise and show large inter-measurement variation [2]. Technical variance in mass spectra can be caused by a number of factors, including the pre-processing of samples in the wet-lab and the mass spectrometer itself. The best way to get maximal power from a statistical analysis is to minimise the level of experimental error and noise. Variance introduced during the pre-processing stages should therefore be minimised as much as possible using strict lab protocols. Furthermore, it makes sense to perform multiple replicate measurements of the same patient. Doing so will give a better estimation of the "true" spectrum of a patient, leading to a better characterisation of the population.

Independent of whether replicate measurements are performed or not, normalisation is usually conducted in order to increase comparability of spectra resulting from different measurements. Normalisation of mass spectra typically entails subtracting an (optional) offset and dividing by a scaling factor. Such offset and scaling parameters can be defined and applied globally, over the full spectrum, or locally, using a sliding window, encompassing a contiguous subset of spectral positions. The rationale behind this is that the global approach may be better able to capture the general characteristics of the data, whereas the local approach may be beneficial for spectra with varying (mass-dependent) noise levels.

Although extensive effort has been devoted towards understanding and modeling the noise [3,4] and variation [5-7] observed in mass spectra, it is unclear which normalisation method is most favourable. Here we perform an extensive comparison of 16 normalisation methods. Please refer to the Methods section for an overview of the methods evaluated. In a comparison of normalisation methods one should employ a measure of the reduction in inter-spectra variation after normalisation to assess the performance of different normalisation methods. However, this may actually promote degradation of the signal in case normalisation entails dividing the original spectrum by a quantity that is proportional to itself. In such a case, the resulting coefficient of variance will be low, although the amplitude of the remaining signal, will, of course, be very low as well; a situation that is clearly highly undesirable.

SELDI and MALDI are typically used for proteome profiling with the goal of finding biomarkers capable of discriminating between different classes of patients, for instance between healthy controls and cancer variants. To accomplish this, various classification techniques can be used to find (sets of) peaks with maximal discriminatory power. To safe-guard the presence of signal after normalisation, in this paper, classification performance is an important measure to compare normalisation methods, in addition to spectral variance. With the above in mind, we define a good normalisation method to be one that adheres to the following objectives:

1. minimises the variance between spectra;

2. maximises the classification signal, i.e., the association of spectra with their respective class labels.

To assess the first objective, the coefficient of variance between spectra is used. The second objective is measured using the performance of three classifier, or "classifier-like", systems. These are the Globaltest, the Support Vector Machine (SVM) with a radial basis kernel and a decision tree (CART).

Note that we assess both objectives separately, yielding two performance indicators, instead of combining them to yield one objective score. The reason for this is that the used objectives are partly conflicting, in the sense that a reduction in variance does not neccessarily lead to an improvement in classification performance. This makes it non-trivial to combine them in a straight-forward manner and forces one to attach weights to each of them, as also illustrated in Additional file 1. Instead of choosing these weights ourselves, we prefer to let the reader decide which of the two objectives is most important.

## Results and Discussion

For this comparison, four different real-world datasets representing two-class diagnostic problems have been analysed, before and after normalisation. For robustness, the pre-processing of spectra was performed using 42 different configurations, the product of seven methods for baseline correction and six peak detection approaches. Normalisation of spectra using the 16 methods under study was carried out after baseline correction and prior to peak detection.

For each of the 42 configurations, both objectives, i.e., inter-spectra variance minimisation and classification performance maximisation, were assessed for each of the normalisation methods as well as for unnormalised data. This results for each normalisation method in two vectors of (ranked) scores, one for each objective, across all 42 pre-processing approaches and four datasets. Please refer to the Methods section for a detailed explanation of how these vectors (Equations 6 and 7) are obtained.

**Figure 1:**
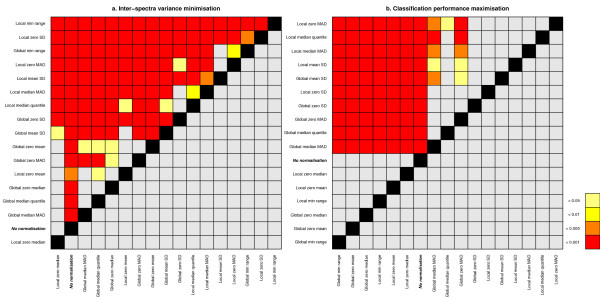
**Relative differences between methods in terms of both objectives**. Plot indicating relative differences between methods in terms of **a**. inter-spectra variance minimisation and **b**. classification performance maximisation. A coloured cell indicates that the ranked scores of the method in the row are significantly better than those of the method in the column, with the colour of the cell corresponding to the order of magnitude of the p-value obtained using paired Wilcoxon signed rank tests. Rows are sorted by the number of coloured cells, giving an indication of relative score differences between methods.

We used non-parametric paired Wilcoxon signed rank tests on these vectors to compare all normalisation methods in terms of the two objectives. Paired tests have been used to control for effects from the baseline correction methods, peak detection approaches and datasets used. We used one-tailed tests to obtain information on relative differences in scores between any two normalisation methods, i.e., whether one method is better than the next, an indicator of the performance of one method versus another. Using two-tailed tests would only provide us with information on whether they perform significantly different. Figure 1 depicts the results of this analysis, for both of the objectives. Some methods perform consistently poorly for both objectives (e.g., "Local zero median"). Others show a large difference in performance between the two objectives. A good example of this is the "Local min range" method, which reduces the variance between spectra more efficiently than any other method, however this has consequences for the classification performance, which is very poor compared to other methods. This illustrates the shortcoming of exclusively minimising the inter-spectra variance and the need for a second objective to safe-guard signal quality. However, note that this second objective should also not be used exclusively, because it is biased towards the classification methods employed and is dependent on the actual datasets used, unlike the inherently unbiased first objective.

### Comparison to no normalisation

**Figure 2:**
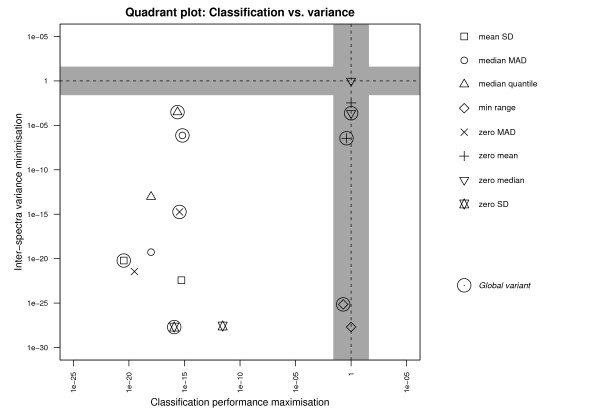
**Significance of the difference in objective scores**. Quadrant plot showing the significance of the difference in objective scores between each normalisation method and unnormalised data. Each normalisation method is represented by a different symbol, global variants being annotated with an additional circle. The axes represent p-values of the statistical analysis described earlier, for classification performance and variance reduction on the x-axis and y-axis, respectively. The point (1, 1) indicates the position of unnormalised data. The region in which a method lies represents its performance for each objective with respect to using no normalisation.

Another way of looking at these results is by comparing all normalisation methods to the case where no normalisation is performed, i.e., the columns indicated by 'No normalisation' in Figure 1. Figure 2 contains a *quadrant plot*, illustrating the performance of each normalisation method against unnormalised data in terms of both objectives, in more detail. The lower left area indicates improvement in both objectives, the lower right area improvement in variance reduction but a deterioration in classification performance and the upper right area a deterioration in both objectives. The shaded bands along the lines where *p *= 1 indicate regions where *p *≥ 0.05, i.e., where differences in scores between the methods in these regions and using unnormalised data are not significant.

As becomes apparent from the figure, the majority of normalisation methods lie within the (non-shaded) lower left region, indicating that they significantly outperform the case when no normalisation is used. An interesting observation here is that the default normalisation method used by most SELDI users ("Global zero mean") slightly reduces the variance but does not improve classification performance significantly with respect to unnormalised data.

### Per-classifier performance

**Figure 3:**
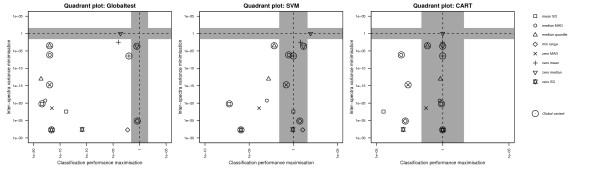
**Significance of the difference in objective scores, for each classifier separately**. Quadrant plots showing the significance of the difference in objective scores, for each classifier separately, between each normalisation method and unnormalised data. **a**. Globaltest, **b**. SVM, **c**. CART.

In order to study what the contribution of individual classifiers is to the overall results, we repeated the statistical analysis for each classifier separately. Since the classifiers employed were chosen from a wide range of classification modalities, this analysis will yield insight into the effect of the choice of classifier on the overall ranking of the methods. The results are depicted in Figure 3. We notice that some normalisation methods cause signal degradation for SVM, placing them in the lower-right area of the quadrant, albeit not significantly worse than employing no normalisation. We further notice that none of the methods perform significantly better than using no normalisation for *all *classifiers. This is largely due to the CART classifier, which has most of the normalisation methods in the (non-significant) shaded areas. As indicated by the range of p-values on the x-axes of the quadrant plots, the increase in classification performance due to normalisation is much lower for CART than it is for the Globaltest and SVM, suggesting that CART is less sensitive to normalisation or is simply incapable of finding the 'good' features, and can therefore not exploit the features where normalisation does show an effect. A paired one-tailed Wilcoxon signed rank test on the raw, i.e., unranked, cross validation classification errors yielded by SVM and CART reveals that SVM performs significantly better than CART overall (*p *= 2.05 × 10^-67^), suggesting the latter.

### Global vs. local normalisation methods

The normalisation methods we studied employ two parameters, an offset and a scaling parameter. The methods can be divided into two groups, depending on whether they are based on global or on local estimates of characteristics of the data. To study whether this parameter (global vs. local) has a significant effect on the two performance objectives, we compared the performance of these two groups of methods. This was done by pairing the performance ranks obtained for local methods with those of their global counterparts, e.g., "Local zero mean" was paired with "Global zero mean", for each of the two objectives. We then used the Wilcoxon signed rank test to test the null-hypothesis that the number of cases in which a method from one group outperforms its paired method from the other group is equal to the number of cases in which the opposite is the case.

**Figure 4:**
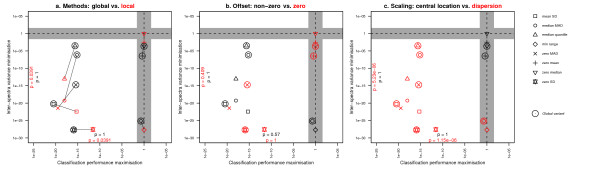
**Detailed analyses of different subgroups of methods**. Quadrant plots showing more detailed analyses of different subgroups of methods. Elements are coloured by group; black vs. red, as indicated in the figure titles. Black p-values indicate the significance with which "black" methods outperform the "red" methods and red p-values the opposite. Lines between elements indicate a pairing of variables for the statistical tests used. The following groupings are used: **a**. global vs. local estimations of normalisation parameters, **b**. non-zero vs. zero offsets and **c**. scaling using the central location vs. the dispersion of data.

We used one-tailed tests to be able to assess which of the two groups performed statistically significantly better. This resulted in two p-values per objective, which were corrected for multiple testing using the Bonferroni method. Figure 4a shows these values, also indicating the used pairings between local and global methods. Global methods are shown in black and local methods in red. We see that for both objectives local methods provide an advantage over global methods (p = 0.0391). Note that this may not be obvious from looking at Figure 4a alone, particularly for the classification performance, because the placement of methods in this figure indicates the performance relative to using no normalisation.

### Offset and scaling parameters

A number of the normalisation methods studied use a zero-valued offset parameter. To study the influence of this on the performance, we again divided the methods into two groups, i.e., methods that employ a non-zero parameter versus those for which this parameter has been set to zero. We used a two-sample Wilcoxon rank sum (i.e., Mann-Whitney U) test to test the null-hypothesis that the two groups outperform the case in which no normalisation is performed, an equal number of times. As before, we tested both tails separately for both objectives, resulting in four p-values, again corrected for multiple testing using the Bonferroni method. Figure 4b shows these values, indicating no significant effect for either of the two objectives.

The same analysis was done for the scaling parameter, which can also be divided into two groups; one containing methods that use the central location of the data (i.e., mean or median) and another containing methods that use some measure of dispersion of the data around that central location (i.e., standard deviation, median absolute deviation or (quantile) range). Figure 4c shows that there is a significant difference here; in terms of both variance reduction (*p *= 5.23 × 10^-6^) and classification performance (*p *= 1.15 × 10^-6^) it is beneficial to choose a scaling parameter based on the dispersion of the data.

## Conclusion

We have performed a systematic comparison of 16 normalisation methods for (SELDI-TOF) mass spectrometry data. For robustness, a large number (42) of configurations for baseline correction and peak detection methods was used, as well as multiple datasets. We used two objectives to assess the benefit of applying a particular normalisation method, namely minimisation of inter-spectra variance and maximisation of classification performance. The latter has proven to be very helpful in safe-guarding against methods that reduce the variance between spectra but at the same time do not improve, or even worsen, the amount of meaningful signal left after normalisation.

We have shown that in the general case, normalisation of mass spectra is beneficial to both objectives; most methods we compared performed significantly better than the case in which no normalisation was used. We have shown that normalisation methods that scale spectra by a factor based on the dispersion (e.g., standard deviation) of the data clearly outperform those where a factor based on the central location (e.g., mean) is used. Additional improvements in performance are obtained when these factors are estimated locally, using a sliding window within spectra, instead of globally, over full spectra. The underperforming category of methods using a globally estimated factor based on the central location of the data includes the method used by the majority of SELDI users, i.e., "Global zero mean".

## Methods

### Normalisation methods

Mass spectra typically exhibit an artefact in the form of a baseline drift, which can be removed by estimating and subtracting it. Such baseline corrected spectra, denoted by *S*, are the basis for normalisation. Normalisation involves applying spectrum specific transformations to *S*. To properly describe normalisation methods, resulting in a normalised spectrum *N*, we define the following model:

(1) $$N=\frac{S-\Theta }{\Delta },$$

where Θ and Δ denote offset and scaling parameters respectively. Both of these parameters can be estimated *globally *over a full spectrum or *locally *using a sliding window, which encompasses a contiguous subset of spectral positions. In the former case, Θ and Δ assume scalar values, while in the latter case, Θ and Δ are vectors of length |*S*|, i.e., the number of measurement points in the spectrum.

Table 1 contains an overview of the normalisation methods evaluated in this study, expressed in terms of combinations of offset and scaling parameters. These have been chosen based on methods described in the literature and on pragmatic reasoning. Methods already used in the field of mass spectrometry have been complemented with more generic methods, including simple variance standardisation.

**Table 1:** Normalisation methods used in the comparison. Normalisation methods used in the comparison. Offset and scaling parameters are used as in Equation 1. Note that for each method these parameters are estimated globally as well as locally and evaluated separately. Formal definitions of these methods can be found in Additional file 2.

**No**	**Offset (Θ)**	**Scaling (Δ)**	**Working name**	**Citation**
1	0	mean	**Zero mean**	[15,16]
2	0	median	**Zero median**	[8]
3	0	sd	**Zero SD**	
4	0	mad	**Zero MAD**	
5	mean	sd	**Mean SD**	
6	median	mad	**Median MAD**	
7	median	iqr	**Median quantile**	[17]
8	min	range	**Min range**	([18])

The last column of Table 1 contains, when possible, citations of studies in which the normalisation methods were used. Note that for each method the global and local variants have been used, resulting in a total of 16 methods. Additional file 2 contains formal definitions of the normalisation methods.

The first method in Table 1 ("zero mean") is a derivation of the method as implemented in the software supplied with SELDI-TOF-MS machines. Strictly speaking, this software (Ciphergen^© ^ProteinChip Software 3.1) uses the mean intensity per spectrum to scale individual spectra and subsequently re-scales all spectra by the mean intensity across all spectra. Because this second scaling is done using a constant scale factor for all spectra, it can easily be left out, as we do here. Although this second scale factor is constant for one normalisation run, it is completely dependent on all spectra, i.e., on their means. This makes it hard to introduce new samples at a later stage or test any built classifier on another dataset. For this reason we only focus on normalisation factors calculated per single spectrum.

As an alternative to using the mean intensity as a scale factor, the median can be used (i.e., method "zero median"). Some studies [8] suggest this may be more robust against outlying peaks.

Another way of normalising spectra is by using the data dispersion, such as the standard deviation (methods "zero SD" and "mean SD"), the median absolute deviation (methods "zero MAD" and "median MAD") or ranges of the data. Method "min range" essentially re-scales the data to the interval [0, 1], either globally or in a local window. As a more robust alternative to this, method "median quantile" uses quantiles of the data.

The citation for method "min range" is parenthesised in Table 1 because it does not strictly follow the normalisation described. In that particular study, normalisation is performed over random subsets of (binned) intensity values instead of over all values (globally) or sliding windows of adjacent values (locally).

### Objective 1: inter-spectra variance

Given the linear relationship between means and standard deviations of peaks (illustrated in last section of Additional file 3), a reasonable measure to compare inter-spectra variation between methods is the coefficient of variation (CV). Defined as the ratio between the standard deviation and the mean $\left(\text{CV}=\frac{s}{\mu }\right)$ it represents a more scale-independent estimation of the variance. However, it may also yield very unstable results for mean values around zero. For this reason, we use an alternative way of computing the CV, adopted from [8]:

(2) $$\text{CV}=\frac{\sum_{i}s_{i}\mu_{i}}{\sum_{i}\mu_{i}^{2}},$$

which is essentially the slope of a regression line through the origin in a scatter plot of s vs. *μ*, such as shown by the dashed line in the figure in Additional file 3.

A sensible way to estimate inter-spectra variance after normalisation would be to inspect the CV calculated over a number of artificially spiked-in peaks and between same-sample replicates. In (existing) real-world datasets however, technical replicates may not be present, let alone artificial spiking peaks. We hypothesised that the CV calculated over all peaks and all available spectra (from all classes) should be a reasonable approximation of the average same-sample replicate CV calculated over spiking peaks only. This is indeed the case, proven by a high Pearson correlation (0.98) between the CVs calculated over the four spiking peaks and over all peaks present in the spectra of this dataset. For this reason, it is justified to use the CV calculated over all peaks. This allows us to use multiple datasets in our comparison of normalisation methods, including datasets lacking spiked-in peptides and/or technical replicates.

### Objective 2: classification performance

In order to assess class separation, and more specifically how it changes under the influence of different normalisation methods, we employ a number of classifiers or classifier-like systems. We employed a single classifier at a time in combination with the variance objective. All classifier analyses were performed using R and various packages that are available for it, and below we elaborate on each of the employed systems.

#### The Globaltest

The Globaltest [9] tests the association of groups of features with a given outcome. Simply put, it tests whether the normalized sum of the correlation coefficients resulting from correlating the peak heights and the class label is sufficiently high to reject the null hypothesis that there is no association between the peak heights and the class label. It is not a classifier, in the sense that it does not output a mapping to assign new samples to one of the outcome classes. It does, however, give an indication of the quality of a dataset with respect to class separation. On top of that, it makes minimal assumptions about the data and has no parameters to be set. The result of using the Globaltest is a single p-value for each group of features, where a group can be defined as all features in a dataset or a subset thereof. Here, we apply this test to all peaks found in a normalised mass spectrum. The globaltest package was used as an implementation of the Globaltest.

#### Support Vector Machine (SVM) with radial basis kernel

A SVM [10] is a classifier that is widely used because of its generally good performance in complex classification problems and especially in applications, such as the problem being studied here, where the number of features is larger than the total number of samples. In essence, it is a classifier in linear space, however transformation to a non-linear space is achieved by using a non-linear kernel, such as the radial basis kernel, as we do here. We used the implementation provided by the e1071 package. To assess the performance of this classifier we use the leave-one-out cross validation error. This was implemented using the ipred package.

#### Classification And Regression Trees (CART)

CART [11] is an algorithm that is widely used within the SELDI community because it is implemented in the software that comes with the mass spectrometer itself and allows for easy interpretation of results. Trees were obtained by a two-step process, making use of the rpart package. Initially, large trees are grown using the Gini index for node impurity. Resulting trees are then, again using a leave-one-out cross validation approach, pruned back to the number of nodes at which the improvement in fit is not significant anymore. This process has the goal of avoiding overfitting. Like with the SVM, we employ the leave-one-out cross validation error to assess the performance of built classifiers.

## Datasets

For robustness, the comparison of normalisation methods has been performed using a total of four SELDI datasets.

• The first dataset is the result of an experiment designed and executed specifically for this study. It is the dataset used to study the relation between mean and standard deviation of peak intensities. For this dataset, real-world serum samples from patients suffering from colorectal cancer (four samples) and controls (four samples) have been artificially spiked with additional peptides. For each sample a total of 10 technical replicates was obtained, yielding a total of 80 spectra, 40 per class. Please refer to Additional file 3 for a more elaborate discussion on (the preparation of) this dataset.

• The second and third datasets resulted from an earlier study [12] concerned with finding biomarkers in human serum to differentiate between colorectal cancer patients and controls. The datasets consist of SELDI measurements of serum samples from 37 colorectal cancer patients vs. 31 controls and 40 colorectal cancer patients vs. 49 controls. Detailed information on the used experimental setup and pre-processing methods can be found in [12].

• The fourth dataset is from the public repository of the Critical Assessment of Microarray Data Analysis (CAMDA) conference 2006 [13]. In this case, the dataset is the result of measuring serum samples of patients suffering from chronic fatigue syndrome as well as control persons, with 62 and 64 samples in each class, respectively. Specifically, the second liquid chromatography fraction of the CM10 CAMDA dataset was used. The used pre-processing protocols can be obtained from the CAMDA website.

### Spectrum pre-processing

All pre-processing of spectra was done using the R statistical software with the MASDA R-package. Pre-processing includes correcting the baseline effect of raw spectra, normalisation and peak detection, filtering and clustering.

We used seven different methods of correcting the baseline of raw spectra before normalisation. These methods include the one implemented in the PROcess R-package, the method used by the manufacturer of SELDI machines and methods based on various smoothing approaches.

After normalisation, peaks are detected in individual spectra, effectively by detecting changes in the first derivative of the intensity curve. This is followed by a process in which peaks are filtered by using estimated noise thresholds, depending on the parameter settings. During the comparison, six different parameter settings were used for robustness. These settings ranged from using no filtering at all to filtering out all peaks below a local noise threshold estimated by a robust local estimator plus five times its median absolute deviation (MAD).

Please refer to Additional file 4 for a more detailed description of the baseline correction and peak detection methods used.

After peak detection and filtering, peaks from different spectra are clustered together using complete linkage hierarchical one-dimensional clustering with a configurable, mass dependent, cut-off point. By default, this point is 0.3% of the peak *m*/*z *value. This mass dependent value is turned into a constant by log10 transforming the *m*/*z *values of detected peaks. For the default value of 0.3%, this yields a cut-off point roughly equal to 0.0013. Resulting peak-clusters, containing one peak per processed spectrum, are used as units in subsequent analyses.

The MASDA mass spectrometry data analysis R-package is freely available [14]. All code to process the datasets is included as Additional file 5.

### Ranked score vector calculation

**Figure 5:**
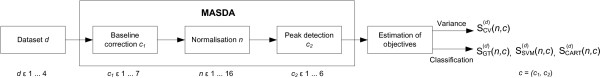
**Schematic representation of the computational workflow**. Schematic representation of the computational workflow used for the comparison. Baseline correction, normalisation and peak detection are implemented in the MASDA R-package. Both objectives used for the comparison are estimated for each dataset *d*, normalisation method *n *and configuration *c *of baseline correction and peak detection parameters. For each tuple (*d*, *n*, *c*), this results in a score ${\text{S}}_{\text{CV}}^{\left(d\right)}$(*n*, *c*) for variance and ${\text{S}}_{\text{GT}}^{\left(d\right)}$(*n*, *c*), ${\text{S}}_{\text{SVM}}^{\left(d\right)}$(*n*, *c*), and ${\text{S}}_{\text{CART}}^{\left(d\right)}$(*n*, *c*) for classification performance.

**Figure 6:**
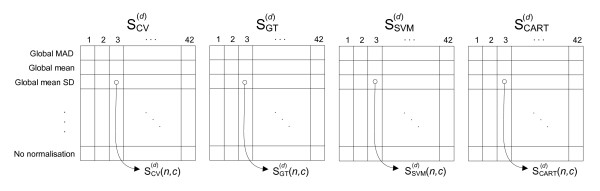
**Structure of tables containing scores resulting from different measurements**. Structure of tables containing scores resulting from different measurements on a dataset *d*, i.e., estimations of the coefficient of variance (${\text{S}}_{\text{CV}}^{\left(d\right)}$) Globaltest p-values (${\text{S}}_{\text{GT}}^{\left(d\right)}$) and leave-one-out cross validation errors for SVM (${\text{S}}_{\text{SVM}}^{\left(d\right)}$) and CART (${\text{S}}_{\text{CART}}^{\left(d\right)}$). Each table consists of 17 rows, representing the 16 normalisation methods (global and local variants) and 'no normalisation'. There are 42 columns in each table, one for each configuration of baseline correction (7) and peak detection (6) approaches.

Figure 5 gives an overview of the workflow used to obtain objective scores for all datasets, normalisation methods and configurations of pre-processing approaches. We employed seven different methods for baseline correction and six different peak detection approaches, combining them results in 42 configurations. For each pre-processing configuration *c *and normalisation method *n *we obtained an estimate of the coefficient of variation and three classification scores (i.e., for Globaltest, SVM and CART). Figure 6 shows the structure of the resulting score tables for each of these four cases, for one dataset d. Note that we obtain such a set of score tables for each dataset used. Also note that we added an extra reference normalisation method entitled "No normalisation", which allows a comparison of the 16 normalisation methods with the case where no normalisation is performed.

Each score table ${\text{S}}_{\text{X}}^{\left(d\right)}$ is converted into a rank table ${\text{R}}_{\text{X}}^{\left(d\right)}$ of identical dimensions by calculating column-wise ranks, i.e., normalisation methods are ranked per configuration *c *of baseline correction and peak detection methods. More formally:

(3) $$\{{\text{R}}_{\text{x}}^{\left(d\right)}(i,c)|i\in 1...17\}=\text{Rank}(\{{\text{S}}_{\text{x}}^{\left(d\right)}(i,c)|i\in 1...17\}).$$

To assess both objectives, i.e., variance minimisation and class separation maximisation, we define two vectors per normalisation method *n *and dataset *d *containing all relevant ranks:

(4) $$r_{\text{VAR}}^{\left(n,d\right)}=\{{\text{R}}_{\text{CV}}^{\left(d\right)}(n,i)|i\in 1...42\}$$

(5) $$r_{\text{CLASS}}^{\left(n,d\right)}=\{[{\text{R}}_{\text{GT}}^{\left(d\right)}(n,i),{\text{R}}_{\text{SVM}}^{\left(d\right)}(n,i),{\text{R}}_{\text{CART}}^{\left(d\right)}(n,i)]|i\in 1...42\},$$

where [*a*, *b*] represents the concatenation of two row vectors. Final vectors $r_{\text{VAR}}^{\left(n\right)}$ and $r_{\text{CLASS}}^{\left(n\right)}$ containing information from all four datasets are obtained by row-wise concatenation and are the basis for the statistical analysis:

(6) $$r_{\text{VAR}}^{\left(n\right)}=[r_{\text{VAR}}^{\left(n,d\right)}|d\in 1...4]$$

(7) $$r_{\text{CLASS}}^{\left(n\right)}=[r_{\text{CLASS}}^{\left(n,d\right)}|d\in 1...4].$$

We use a non-parametric paired Wilcoxon signed rank test to compare two normalisation approaches, separately for the two objectives. Tests are performed on all possible pairings of normalisation methods, to assess whether rankings are significantly different between methods, irrespective of the used baseline correction method, peak detection approach and dataset. More specifically, we use one-tailed tests in order to obtain information on relative performance differences between normalisation methods. Bonferroni multiple testing corrected p-values were obtained by multiplying the raw p-values with a correction factor equal to the number of normalisation methods employed, i.e., 17.

All code to analyse the datasets as described above, and to exactly generate the figures used in this paper, is included as Additional file 6.

### Construction of quadrant plots

Quadrant plots (Figures 2, 3 and 4) aim to visualise the performance of normalisation methods, simultaneously for the two objectives. For each objective, we use two one-tailed tests to assess whether the performance of a particular normalisation method is significantly better or worse than when using no normalisation. We employed one-tailed tests, since we wanted to test the directionality of the association, and then correct for multiple testing. Because we are interested in differences between normalisation methods, we only use the lowest of the two p-values obtained from the two one-tailed tests per objective. For each normalisation method, we then plot a symbol representing it in the quadrant plot, using the two resulting p-values (one for each objective) as coordinates.

## Authors' contributions

WM performed the analyses and wrote the manuscript. WM, MJTR and LFAW designed the dry-lab experiments. WM, JYMNE, MCWG and JHB were involved in designing and performing the wet-lab experiment with the spiking peptides. All authors read and approved the final manuscript.

## Supplementary Material

Additional File 1 **Illustration of combined objectives**. PDF-file illustrating the issues around combining the objectives used in this study into one. Click here for file

Additional File 2 **Formal definitions of normalisation methods**. PDF-file containing formal definitions of the normalisation methods included in this study. Click here for file

Additional File 3 **Processing protocol used for spiked dataset**. PDF-file containing an overview of the biological samples, spiking mixture, experiment design and laboratory conditions used to generate the spiked dataset. Also contains an illustration of the relation between the mean intensity of peaks and their standard deviation. Click here for file

Additional File 4 **Baseline correction and peak detection methods**. PDF-file containing descriptions and references to implementations and papers for the various baseline correction and peak detection methods used in the paper. Click here for file

Additional File 5 **R-code for data processing**. ZIP-file containing R-code used to process all datasets with all normalisation methods and parameter configurations. Makes use of the MASDA R-package for Mass Spectrometry Data Analysis. Click here for file

Additional File 6 **R-code for analysis of results**. ZIP-file containing all objective scores for all datasets, normalisation methods and parameter configurations used in the comparison, along with all R-code needed to generate the figures in the paper. Click here for file
