# Supplementary material for: Comparison of normalisation methods for surface-enhanced laser desorption and ionisation (SELDI) time-of-flight (TOF) mass spectrometry data
Source: BMC Bioinformatics. 2008 Feb 7;9:88. doi: 10.1186/1471-2105-9-88 (PMC2258289; doi:10.1186/1471-2105-9-88)
Supplement: Additional File 1 — Illustration of combined objectives. PDF-file illustrating the issues around combining the objectives used in this study into one. [file 1471-2105-9-88-S1.pdf]

A1. Illustration of combined objectives

Combining both objectives requires a model such as for example

$$\psi = -\alpha \log_{10}(p_1) - (1-\alpha)\log_{10}(p_2) \ ,$$

where ψ denotes a final score after combining the p-values p_1 and p_2 of both objectives, inter-spectra variance minimisation (1) and classification performance maximisation (2), respectively, with weighting factor α. This weighting factor dictates the importance of objectives. The score ψ can then be maximised to obtain "the best" normalisation method. Naturally, this depends heavily on the value of α, which depends on user preference. Figure 1 below illustrates this, by way of the two grey lines. Line 1 favours the variance minimisation objective and penalises the classification performance maximisation objective, by having $\alpha > 0.5$, thereby choosing method "Global zero SD" as the best. Line 2, however, favours the classification maximisation objective and penalises the variance minimisation objective, by having $\alpha < 0.5$, yielding "Global mean SD" as being the best method.

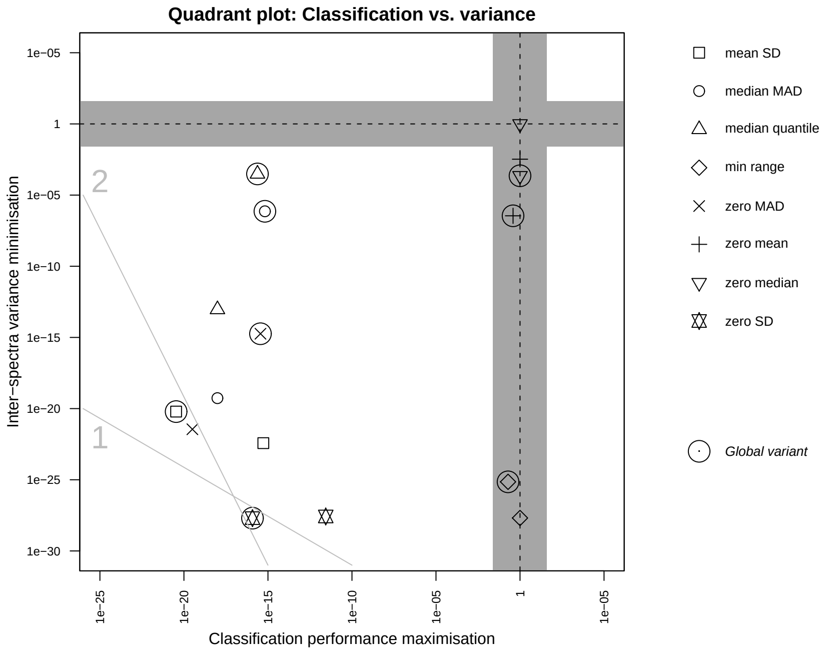

Figure 1: Quadrant plots showing different possibilities of combining the two objectives into one, i.e., the two grey lines.

Another way of looking at the performance of normalisation methods with respect to the case in which no normalisation is used is provided by Figure 2. It contains for each normalisation method (rows) the

Percentage of cases better than ‘No normalisation’

	Globaltest	SVM	CART	Variance	Globaltest & Variance	SVM & Variance	CART & Variance	Classification & Variance
Global zero SD	81	**62**	51	**99**	**80**	**61**	50	**97**
Local zero SD	76	53	60	98	75	52	**58**	95
Local mean SD	80	54	**64**	89	69	46	**58**	86
Global mean SD	**85**	**62**	52	86	74	50	45	84
Global zero MAD	84	57	62	81	67	42	51	79
Local zero MAD	79	58	58	81	69	48	48	78
Global median MAD	**85**	52	62	79	67	39	51	77
Local median MAD	82	61	51	80	68	49	42	77
Local median quantile	82	59	54	74	62	41	42	71
Local min range	61	43	49	98	61	43	49	80
Global median quantile	83	55	57	70	60	35	43	70
Global min range	54	38	61	92	46	32	**58**	80
Global zero mean	60	42	53	64	51	36	36	62
Global zero median	60	42	45	58	41	27	30	53
Local zero mean	58	33	51	46	37	26	24	43
Local zero median	54	35	46	31	26	20	16	29

Figure 2: Percentage of cases in which methods are better than using no normalisation method. Rows indicate the 16 normalisation methods, columns indicate different criteria, either on their own or combined. The best percentages in each column are indicated in boldtype and rows are ordered by their rowsum.

percentage of cases in which it performed better than using no normalisation, in terms of certain criteria (columns). The last column indicates the percentage of cases in which *at least one* classifier outperformed “No normalisation”, in addition to an improvement in variance.

Rows in the figure are ordered by their rowsum, i.e. the overall percentage over all criteria. What is interesting in view of this ordering is the order of normalisation scaling parameters. Methods using the standard deviation (SD) as a scaling parameter are structurally found at the top, followed by methods using the median absolute deviation (MAD). The methods employing mean and median as scaling parameter are all at the bottom.

However, we have to stress that, again, this method of combining the two objectives depends heavily on user preference. We therefore leave these choices to the reader.
