# Supplementary material for: Comparison of normalisation methods for surface-enhanced laser desorption and ionisation (SELDI) time-of-flight (TOF) mass spectrometry data
Source: BMC Bioinformatics. 2008 Feb 7;9:88. doi: 10.1186/1471-2105-9-88 (PMC2258289; doi:10.1186/1471-2105-9-88)
Supplement: Additional File 2 — Formal definitions of normalisation methods. PDF-file containing formal definitions of the normalisation methods included in this study. [file 1471-2105-9-88-S2.pdf]

A2. Formal definitions of normalisation methods

Normalisation of a baseline corrected spectrum S results in a normalised spectrum N, defined as

$$N = \frac{S - \Theta}{\Delta},$$

where Θ and Δ denote offset and scaling parameters respectively. Both of these parameters can be estimated *globally* over a full spectrum or *locally* using a sliding window, which encompasses a contiguous subset of 1000 spectral positions. In the former case, Θ and Δ assume scalar values, while in the latter case, Θ and Δ are vectors of length $|S|$, i.e., the number of measurement points in the spectrum.

Table 1 in the main document contains informal definitions of the normalisation methods used in this study. More formal definitions of offset and scaling parameters are provided below, where $X = \{x_i \,|\, i \in 1 \ldots n\}$ a vector of length $n = |S|$ or $n = 1000$, depending on whether a global or local normalisation method is used. In case of the former, $X = S$ and parameter values are scalar. In case of the latter, parameters are calculated using a sliding window of size 1000, yielding final offset and scaling parameter values of length $|S|$.

$$\begin{aligned}
\text{mean}(X) &= \frac{1}{n}\sum_{i=1}^{n} x_i \\
\text{median}(X) &= Q_{.50}(X) \\
\text{sd}(X) &= \frac{1}{n-1}\sum_{i=1}^{n} (x_i - \text{mean}(X))^2 \\
\text{mad}(X) &= Q_{.50}\left(|X - Q_{.50}(X)|\right) \\
\text{iqr}(X) &= Q_{.75}(X) - Q_{.25}(X) \\
\text{range}(X) &= Q_1(X) - Q_0(X),
\end{aligned}$$

where Q_p is defined as follows:

$$\begin{aligned}
Q_p &= (1-h)u_i + hu_{i+1} \\
h &= p(n+1) - \lfloor p(n+1) \rfloor \\
i &= \max\{\min\{\lfloor p(n+1) \rfloor, n\}, 1\}) \\
U &= \text{sort}(X),
\end{aligned}$$

where $\text{sort}(X)$ transforms X into U, such that $\{u_1 \leq u_2 \leq \ldots \leq u_n\}$.

All these methods are implemented in the MASDA R-package for mass spectrometry data analysis.
