# Supplementary material for: Comparison of normalisation methods for surface-enhanced laser desorption and ionisation (SELDI) time-of-flight (TOF) mass spectrometry data
Source: BMC Bioinformatics. 2008 Feb 7;9:88. doi: 10.1186/1471-2105-9-88 (PMC2258289; doi:10.1186/1471-2105-9-88)
Supplement: Additional File 3 — Processing protocol used for spiked dataset. PDF-file containing an overview of the biological samples, spiking mixture, experiment design and laboratory conditions used to generate the spiked dataset. Also contains an illustration of the relation between the mean intensity of peaks and their standard deviation. [file 1471-2105-9-88-S3.pdf]

A3. Processing protocol used for spiked dataset

To study the behaviour of the variation of peaks between spectra with respect to their mean intensity we performed an experiment in which a number of real-world serum samples have been artificially spiked with four peptides of known concentration. Here, the used biological samples, spiking mixture, experiment design and laboratory conditions used are discussed.

Sample pre-processing

Spiking mixtures were freshly prepared from individual peptides (Ciphergen Biosystems Inc., Freemont, CA, USA). A 100 μl-stock solution containing a mixture of dynorphin (2147.5 Da), ACTH 1-24 (2933.5 Da), β-endorphin (3465.6 Da) and ubiquitin (8564.8 Da), each 1 nmol/100 μl, in deionised water was prepared. In pilot experiments the optimal dilution for spiking in serum was assessed, resulting in an optimal dilution of 1:300 when applying 20 μl of sample to the chip.

Serum samples used for the experiment have been obtained from patients and healthy controls who are all men. Means (and standard deviations) of ages for patient and control groups are 66.65 (17.10) and 68.54 (16.52), respectively. All samples have been obtained during the same period of two months and have been frozen at $-80°$. Before the experiment, samples were thawed and denatured by adding 190 μl of a solution of 9M urea and 2% CHAPS, (Sigma, St. Louis, MO, USA) to 10 μl of serum. Spiked sera were prepared by adding 10 μl of a 1:15 dilution of spiking solution to 10 μl of serum and 180 μl of urea 9M/CHAPS 2%. As energy absorbing matrix a 50% solution of sinapinic acid (SPA; Ciphergen Biosystems) in 50% acetonitrile (ACN) + 0.5% trifluoracetic acid (TFA) was used. Spiked matrix was prepared by adding 6 μl of the 1:15 spiking solution to a total of 400 μl SPA solution.

During all steps of the protocol, the bioprocessor was placed on a platform shaker at 350 rpm. Chips were equilibrated twice with 200 μl of binding buffer for 5 min. Subsequently, 180 μl of binding buffer and 20 μl of denatured sample were applied to the chip surface. Incubation was set to 30 min. After binding, the chips were washed twice for 5 min with binding buffer, followed by two 5-min washes with wash buffer. Lastly, chips were rinsed with deionised water, air-dried and finished with two 1-μl SPA applications to the sample spots.

We performed SELDI-TOF mass spectrometry (Ciphergen Biosystems) with CM 10 chips (weak cation exchange chip containing anionic carboxylate groups that bind positively charged proteins in serum) with a 100 mM sodium acetate (Sigma) binding buffer, pH 4, and a 50 mM HEPES wash buffer.

Measurements

Protein chips were analysed using the PBS-IIC ProteinChip Reader (Ciphergen Biosystems). In accordance with the recommendations of, among others, [1], the following conditions have been used:

1. The instrument was calibrated externally with a standard peptide mixture (Ciphergen Biosystems) containing vasopressin (1084.3 Da), somatostatin (1 637.9 Da), dynorphin (2147.5 Da), ACTH (2933.5 Da), insulin β-chain (bovine; 3495.5 Da), insulin (human recombinant; 5807.7 Da) and hirudin (7033.6 Da).

2. All used chips originated from the same batch.

3. All buffer and matrix material was prepared fresh and in one batch.

4. Each unique sample has been measured in quintuplicate (i.e., five replicates).

5. Replicate measurements were performed on different spots and or chips.

6. All measurements and pre-processing steps are performed by exactly one operator.

Data were collected between 0 and 100000 Da, optimisation range from 1500 to 50000 Da and spot positions 22 to 82. Data collection was optimised for detection of both serum and spiking peptide peaks, resulting in an average of 65 laser shots per spectrum at laser intensity 150, detector sensitivity 5 and laser focusing at 10000 Da. A second data collection was done a week later with spot positions 20 to 80 and laser intensity 155, correcting for any degradation in laser signal. This yielded a total of 10 (5 $\times$ 2 replicate spectra for each sample in the sample set.

In order to detect any chip and/or spot biases, we performed an ANOVA analysis on the peak heights in the unnormalised spectra using chip and spot positions as factors. No systematic biases were discovered (data not shown).

Relation between mean and variation of peak intensity

Figure 1 shows that there exists a strong linear relation between the means and standard deviations of the spiking peaks, indicating that the latter increase with the former. The scales of normalised spectra may vary considerably between normalisation methods, suggesting that the coefficient of variance is a good method to measure variance.

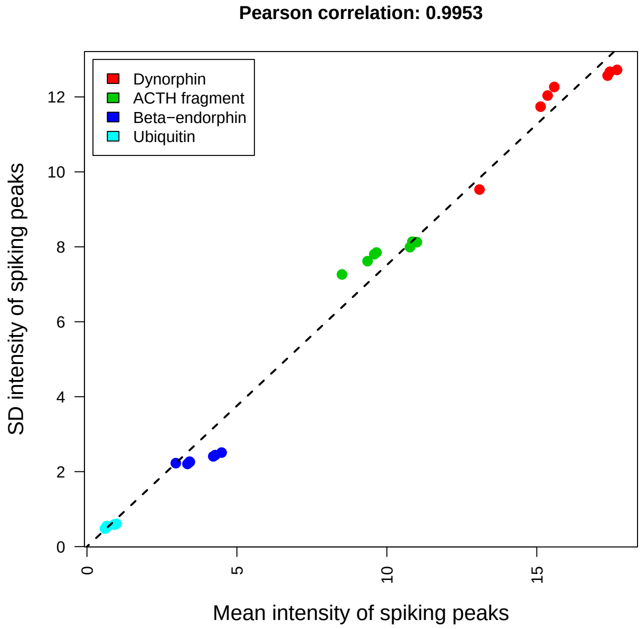

Figure 1: Mean intensities of spiking peaks versus their standard deviation. Same-coloured points indicate the results for different baseline correction methods.

References

1. C. Nicole White, Daniel W. Chan, and Zhen Zhang. Bioinformatics strategies for proteomic profiling. *Clinical Biochemistry*, 37(7):636–641, July 2004.
