# Supplementary material for: Comparison of normalisation methods for surface-enhanced laser desorption and ionisation (SELDI) time-of-flight (TOF) mass spectrometry data
Source: BMC Bioinformatics. 2008 Feb 7;9:88. doi: 10.1186/1471-2105-9-88 (PMC2258289; doi:10.1186/1471-2105-9-88)
Supplement: Additional File 4 — Baseline correction and peak detection methods. PDF-file containing descriptions and references to implementations and papers for the various baseline correction and peak detection methods used in the paper. [file 1471-2105-9-88-S4.pdf]

A4. Baseline correction and peak detection methods

Descriptions and references to implementations and papers for the various baseline correction and peak detection methods used in the paper. All methods are implemented in the MASDA `R`-package.

№	Description	Implementation, citation
1	Varying width segmented convex hull algorithm	Ciphergen© ProteinChip Software, [4]
2	Robust locally weighted regression (LOWESS) with a span $f = 0.01$ and $\delta = 100$	`R`-function `lowess`, [1]
3	Friedman's "super smoother" with a span of 0.01	`R`-function `supsmu`, [3]
4	Cubic smoothing spline	`R`-function `smooth.spline`, [5]
5	Cubic spline interpolation of locally minimal points only	`R`-function `splinefun`, [2]
6	Same as method 5, except result is further smoothed using Tukey's "3RS3R" smoother	`R`-functions `splinefun` and `smooth`, [2, 6]
7	LOESS smoothed low-percentile intensity	`R`-function `bslnoff` in package `PROcess`

Table 1: Baseline correction methods used in the analysis. For each method, the baseline was estimated and then subtracted from the data. Here, we describe the methods of estimation.

№	Description	Implementation, citation
1	No threshold, all peaks are included	
2	Friedman's "super smoother" on intensities	`R`-function `supsmu`, [3]
3	Median intensity plus five times median absolute deviation, both estimated using a sliding window (size = 1000)	
4	Friedman's "super smoother" on result of method 3	`R`-function `supsmu`, [3]
5	Mean intensity plus five times standard deviation, both estimated using a sliding window (size = 1000)	
6	Friedman's "super smoother" on result of method 5	`R`-function `supsmu`, [3]

Table 2: Peak detection methods used in the analysis. For each method, peaks were obtained by detecting sign changes in the slope of the intensity signal, equivalent to a change in the first derivative of a continuous signal. These peaks were then filtered according to a threshold; peaks above it are retained. Here, we describe the methods for setting this threshold.

References

1. William S. Cleveland and Susan J. Devlin. Locally weighted regression: An approach to regression analysis by local fitting. *Journal of the American Statistical Association*, 83(403):596–610, 1988.

2. G. E. Forsythe, M. A. Malcolm, and C. B. Moler. *Computer Methods for Mathematical Computations*. Prentice-Hall, Englewood Cliffs, NJ, USA, 1977.

3. J. H. Friedman. A variable span scatterplot smoother. Technical Report 5, Laboratory for Computational Statistics, Stanford University, 1984.

4. Eric T Fung and Cynthia Enderwick. ProteinChip clinical proteomics: computational challenges and solutions. *Biotechniques*, Suppl:34–8, 40–1, Mar 2002.

5. T.J. Hastie and R.J. Tibshirani. *Generalized Additive Models*. Chapman and Hall, New York, 1990.

6. J. W. Tukey. *Exploratory Data Analysis*. Addison-Wesley, Reading, MA, USA, 1977.
